# Adapting Logic Models Over Time: The Washington State Heart Disease and Stroke Prevention Program Experience

**Published:** 2008-03-15

**Authors:** Susan Ladd, Marilyn Sitaker, Miriam Patanian, Jan Jernigan

**Affiliations:** Behavioral Scientist, Division for Heart Disease and Stroke Prevention, Centers for Disease Control and Prevention; Washington State Department of Health, Olympia, Washington; Washington State Department of Health, Olympia, Washington; Division for Heart Disease and Stroke Prevention, Centers for Disease Control and Prevention, Atlanta, Georgia

## Abstract

Logic models are graphic representations of the relationship between program activities and their intended effects and are used for both program planning and evaluation. Logic models can provide an important foundation for program evaluation by identifying evaluation questions that most appropriately assess program processes and outcomes and by guiding measurement decisions. We demonstrate how logic models can be used to plan program evaluation by describing the adoption of logic modeling by the Washington State Heart Disease and Stroke Prevention Program (WaHDSPP) and by specifying the changes in process and use of logic models since the program's initial funding. Our paper describes how a logic model was used in generating the program evaluation plan for the WaHDSPP, including the identification of evaluation questions and development of indicators to track progress effectively. We describe the use of evaluation results, as well as steps state programs can take to use logic models in program evaluation.

## Introduction

The benefits of logic models for program planning and evaluation are evident. As a result, descriptions of logic models are becoming more commonplace in program and evaluation literature, and funding announcements now routinely encourage their development and use. For example, when the Centers for Disease Control and Prevention's (CDC's) Division for Heart Disease and Stroke Prevention (DHDSP) released its 5-year Funding Opportunity Announcement (FOA) for 2007 for state programs to address heart disease and stroke, applicants were required to develop logic models. However, although application requirements now more commonly require logic models, the subsequent use of these models once grants have been awarded may be inconsistent. We describe the Washington State Heart Disease and Stroke Prevention Program's (WaHDSPP's) experience with program logic models and their use in evaluation planning. We also describe how the development and use of program logic models evolved over time. By describing how the WaHDSPP developed its logic model and used it to construct an evaluation plan, we hope to demonstrate the link between development and use. Subsequently, we hope to demonstrate the utility of logic models in public health programs and stimulate the construction of better and more usable evaluation plans.

## Overview of the WaHDSPP

The goal of the WaHDSPP is to build statewide support for programs targeting people who have heart disease or history of stroke, as well as people who are at high risk for developing either condition (e.g., people with hypertension, people with high blood cholesterol, people with diabetes). The WaHDSPP first received funding from CDC's DHDSP in 2003. From 2003 through 2006, the program was staffed by a full-time program manager and half-time epidemiologist. During this time, the program relied heavily on cross-program staff from the Washington State Department of Health, internal and external partners, and the WaHDSPP advisory council to implement its activities.

## History of Logic Model Use by the WaHDSPP for Planning and Evaluation

Logic models were not a requirement for funding by CDC, and during the first 2 years (2003–2004) of the program, no logic model existed to describe or assist in evaluating the WaHDSPP. Activities conducted by the WaHDSPP were chosen on the basis of their alignment to CDC DHDSP program priorities, the existing program capacity, relationships with internal and external partners, and previous experience. During this time, the WaHDSPP focused on capacity building and needs assessment activities, including developing *The Burden of Heart Disease and Stroke in Washington State* (1) (referred to hereafter as "the Burden document"), forming a statewide advisory council, and conducting an environmental scan of extant population resources for prevention and control of heart disease and stroke. Using the CDC DHDSP framework included in *A Public Health Action Plan to Prevent Heart Disease and Stroke* (2) (referred to hereafter as "the Action Plan") and findings from the environmental scan and the Burden document, the advisory council developed the Washington State Public Health Action Plan for Heart Disease and Stroke Prevention and Management (3) (referred to hereafter as "the State Plan"). Because projects had not been implemented and the logical link to expected outcomes of these capacity activities had not been identified through a logic model, the WaHDSPP did not engage in any program evaluation activities during 2003–2004.

**Figure 1.**
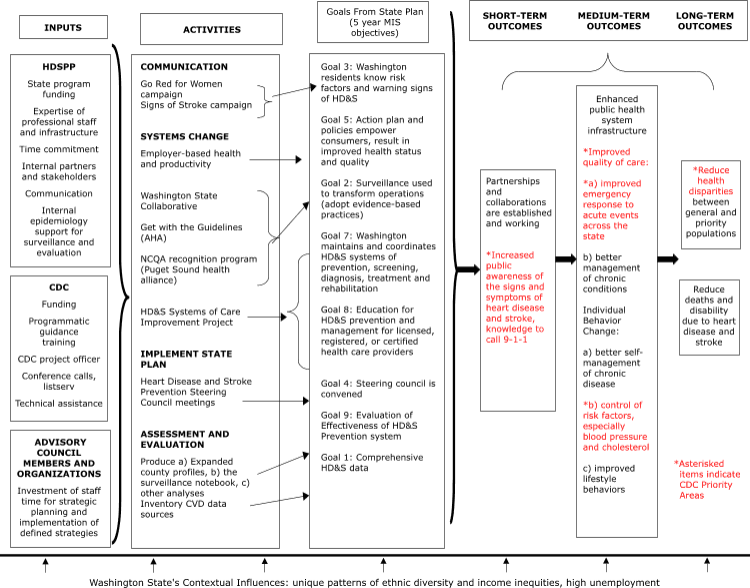
Washington State Specific Logic Model Framework for Heart Disease and Stroke Prevention Program. (HDSPP indicates Heart Disease and Stroke Prevention Program; CDC, Centers for Disease Control and Prevention; and HD&S, heart disease and stroke.)

Although not a requirement for continued funding, logic model use was encouraged by the DHDSP to assist in program planning and evaluation, and the DHDSP provided training and technical assistance to states for the development and use of logic models. Recognizing the benefits of logic models, the WaHDSPP developed a simple logic model that described its planned activities and expected outcomes as part of the 2005–2006 continuation application. Each activity in the 2005–2006 logic model (Figure 1) was linked to an essential public health service objective from the WaHDSPP State Plan. However, as the only staff members for the WaHDSPP, the program manager and epidemiologist produced the logic model in isolation, with no input from cross-program staff or partners.

**Table:** 

The figure begins with a series of boxes organized into three columns. The three boxes arranged on top of each of the columns read 1) Inputs, 2) Activities, and 3) Goals From State Plan (5 year MIS objectives). In the first column, entitled “Inputs,” are three boxes arranged from top to bottom. Inside the first box, entitled “HDSPP,” are the following bullet points: state program funding; expertise of professional staff and infrastructure; time commitment; internal partners and stakeholders; communication; and internal epidemiology support for surveillance and evaluation. Inside the second box, entitled “CDC,” are the following bullet points: funding; programmatic guidance; training; CDC project officer; conference calls, listserv; and technical assistance. Inside the third box, entitled, “Advisory Council Members and Organizations,” is the following information: investment of staff time for strategic planning and implementation of defined strategies. All three boxes in the first column are connected with a right-pointing bracket to the boxes in the second column.
The second column, entitled “Activities,” contains one large box beneath the title box. The large box contains four major sections, each with information listed beneath. The four major sections are: 1) Communication, 2) Systems Change, 3) Implement State Plan, and 4) Assessment and Evaluation. Underneath the “Communication” section is the following information: Go Red for Women campaign; and Signs of Stroke campaign. Underneath the “Systems Change” section are the following bullet points: employer-based health and productivity; Washington State Collaborative; Get with the Guidelines (AHA); NCQA recognition program (Puget Sound health alliance); and HD&S Systems of Care Improvement Project. Underneath the “Implement State Plan” section is the following information: Heart Disease and Stroke Prevention steering council meetings. Underneath the “Assessment and Evaluation” section is the following information: produce a) expanded county profiles, b) the surveillance notebook, c) other analyses; and inventory CVD data sources.
The third column, entitled “Goals From State Plan (5 year MIS objectives),” contains one large box beneath the title box. The large box contains a listing of eight goals, which are organized in the following order: 1) Goal 3: Washington residents know risk factors and warning signs of HD&S; 2) Goal 5: action plan and policies empower consumers, result in improved health status and quality; 3) Goal 2: surveillance used to transform operations (adopt evidence–based practices); 4) Goal 7: Washington maintains and coordinates HD&S systems of prevention, screening, diagnosis, treatment and rehabilitation; 5) Goal 8: education for HD&S prevention and management for licensed, registered, or certified health care providers; 6) Goal 4: steering council is convened; 7) Goal 9: evaluation of effectiveness of HD&S prevention system; and 8) Goal 1: comprehensive HD&S data.
All of the information in the large box in the second column is connected to the goals listed in the large box in the third column, using right-pointing arrows. “Go Red for Women Campaign” and “Signs of Stroke Campaign” under the “Communication” section of column two is connected to “Goal 3: Washington residents know risk factors and warning signs of HD&S” in column three. “Employer-based health and productivity” under the “Systems Change” section of column two is connected to “Goal 5: action plan and policies empower consumers, result in improved health status and quality” in column three. “Washington State Collaborative,” “Get with the Guidelines (AHA),” and “NCQA recognition program (Puget Sound health alliance)” under the “Systems Change” section of column two are connected to “Goal 2: Surveillance used to transform operations (adopt evidence–based practices)” in column three. “HD&S Systems of Care Improvement Project” under the “Systems Change” section of column two is connected to “Goal 7: Washington maintains and coordinates HD&S systems of prevention, screening, diagnosis, treatment and rehabilitation” and to “Goal 8: education for HD&S prevention and management for licensed, registered, or certified health care providers” in column three. “Heart Disease and Stroke Prevention steering council meetings” under the “Implement State Plan” section of column two is connected to “Goal 4: steering council is convened” in column three. “Produce a) expanded county profiles, b) the surveillance notebook, c) other analyses” under the “Assessment and Evaluation” section of column two is connected to “Goal 9: evaluation of effectiveness of HD&S prevention system” in column three. “Inventory CVD data sources” under the “Assessment and Evaluation” section of column two is connected to “Goal 1: comprehensive HD&S data” in column three.
To the right of column three are another series of three columns, each with a heading box above them. The first three columns already described are connected to the second series of three columns with a right-pointing bracket. The heading boxes above the second set of three columns read, “Short-term Outcomes,” “Medium-term Outcomes,” and “Long-term Outcomes.” Inside the first box of the second set of columns is the following information: partnerships and collaborations are established and Working; increased public awareness of the signs and symptoms of heart disease and stroke, knowledge to call 9-1-1 [noted as a CDC priority area]. Box one is connected with a right-pointing arrow to box two of the second set of columns. Inside the second box there are three sections. The first section reads, “Enhanced public health system infrastructure.” The second section, entitled “Improved quality of care,” contains the following two bullet points: a) improved emergency response to acute events across the state, and b) better management of chronic conditions. (The entire section of “Improved quality of care” is noted as a CDC priority area.) The third and last section of the second box, entitled “Individual Behavior Change,” contains the following three bullet points: a) better self-management of chronic disease; b) control of risk factors, especially blood pressure and cholesterol [noted as a CDC priority area]; and c) improved lifestyle. Box two is connected with a right-pointing arrow to two separate boxes in column three. These two smaller boxes are arranged vertically to form the final column. Inside the top box of column three is the following information: Reduce health disparities between general and priority populations. (The words “reduce health disparities” are noted as a CDC priority area.) Inside the bottom box of column three is the following information: Reduce deaths and disability due to heart disease and stroke.
Underneath the six columns is the following text: Washington State’s Contextual Influences: unique patterns of ethnic diversity and income inequities, high unemployment. This text has a series of seven arrows that point upwards toward a large bolded line that lies beneath the six columns.

**Figure 2.**
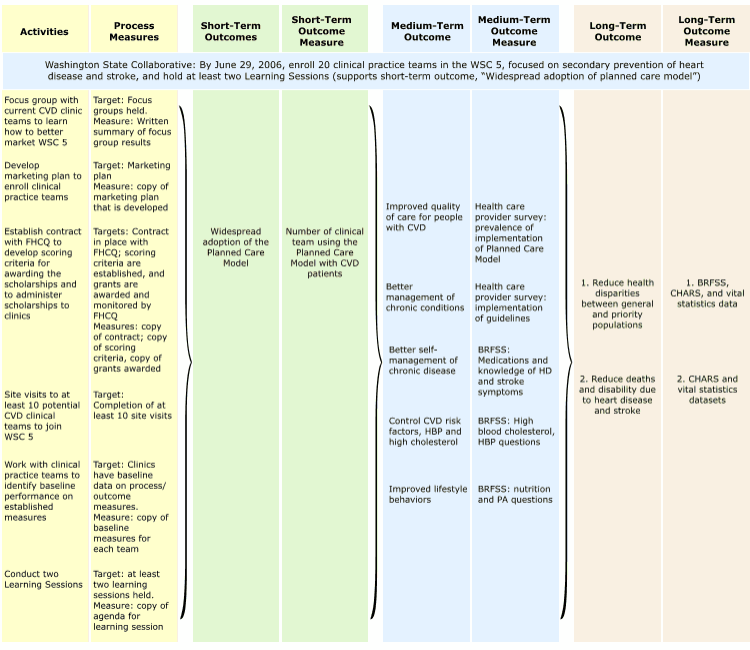
Washington State Heart Disease and Stroke Prevention Program (WaHDSPP) Logic Evaluation Plan, 2005–2006. (WSC indicates Washington State Collaborative; CVD, cardiovascular disease; FHCQ, Foundation for Healthcare Quality; HBP, high blood pressure; BRFSS, Behavioral Risk Factor Surveillance System; HD, heart disease; PA, physical activity; and CHARS, Comprehensive Hospital Abstract Reporting System.)

To assist in evaluation planning, a logic evaluation plan (LEP) was developed in Excel (Microsoft Corporation, Redmond, Washington), linking evaluation measures to each activity and outcome in the logic model. The 2005–2006 LEP for one activity, the Washington State Collaborative, is shown in Figure 2. The evaluation included some process measures, as well as measures of progress toward short-, medium-, and long-term outcomes. The LEP not only provided simple measures for assessing program activities and outcomes, it also helped the WaHDSPP identify and address measurement gaps. For example, to measure the impact of the Washington State Collaborative on the medium-term outcome *better management of chronic conditions*, the WaHDSPP developed and implemented a statewide survey of health care providers to assess implementation of the planned care model by primary care physicians and to assess providers' use of evidence-based guidelines.

**Table:** 

This figure contains a series of boxes arranged into eight columns, each of which contains a title box: 1) Activities, 2) Process Measures, 3) Short-Term Outcome, 4) Short-Term Outcome Measure, 5) Medium-Term Outcome, 6) Medium-Term Outcome Measure, 7) Long-Term Outcome, and 8) Long-Term Outcome Measure. Underneath these eight heading boxes is one long, rectangular box that stretches the length of the figure. The following text is contained in the long box: Washington State Collaborative: By June 29, 2006, enroll 20 clinical practice teams in the WSC 5, focused on secondary prevention of heart disease and stroke, and hold at least two Learning Sessions (supports short-term outcome, “Widespread adoption of planned care model”). Underneath the heading box of the first column, entitled “Activities,” are the following six bullet points: 1) focus group with current CVD clinic teams to learn how to better market WSC 5; 2) develop marketing plan to enroll clinical practice teams; 3) develop marketing plan to enroll clinical practice teams; 4) site visits to at least 10 potential CVD clinical teams to join WSC 5; 5) work with clinical practice teams to identify baseline performance on established measures; and 6) conduct two learning sessions.
Underneath the heading box of the second column, entitled “Process Measures,” are six bullet points. The first bullet point says, “Target: Focus groups held. Measure: Written summary of focus group results.” The second bullet point says, “Target: Marketing plan. Measure: copy of marketing plan that is developed.” The third bullet point says, “Targets: Contract in place with FHCQ; scoring criteria are established, and grants are awarded and monitored by FHCQ. Measures: copy of contract; copy of scoring criteria, copy of grants awarded.” The fourth bullet point says, “Target: Completion of at least 10 site visits.” The fifth bullet point says, “Target: Clinics have baseline data on process/outcome measures. Measure: copy of baseline measures for each team.” The sixth bullet point says, “Target: at least 2 Learning Sessions held. Measure: copy of agenda for learning session.” The first two columns are connected to the third and fourth columns with a right-pointing bracket.
Underneath the heading box of the third column, entitled “Short-Term Outcome,” is the following information: Widespread adoption of the Planned Care Model. Underneath the heading box of the third column, entitled “Short-Term Outcome Measure,” is the following information: Number of clinical team using the Planned Care Model with CVD patients. The third and fourth columns are connected to the fifth and sixth columns with a right-pointing bracket.
Underneath the heading box of the fifth column, entitled “Medium-Term Outcome,” are five bullet points. The first bullet point says, “Improved quality of care for people with CVD.” The second bullet point says, “Better management of chronic conditions.” The third bullet point says, “Better self-management of chronic disease.” The fourth bullet point says, “Control CVD risk factors, HBP and high cholesterol.” The fifth bullet point says, “Improved lifestyle behaviors.”
Underneath the heading box of the sixth column, entitled “Medium-Term Outcome Measure,” are five bullet points. The first bullet point says, “Health care provider survey: Prevalence of implementation of Planned Care Model.” The second bullet point says, “Health care provider survey: Implementation of guidelines.” The third bullet point says, “BRFSS: Medications and knowledge of HD and stroke symptoms.” The fourth bullet point says, “BRFSS: High blood cholesterol, High blood pressure questions.” The fifth bullet point says, “BRFSS: Nutrition and PA questions.” The fifth and sixth columns are connected to the seventh and eighth columns with a right-pointing bracket.
Underneath the heading box of the seventh column, entitled “Long-Term Outcome,” are the following two bullet points: 1) Reduce health disparities between general and priority populations; and 2) Reduce deaths and disability due to heart disease and stroke. Underneath the heading box of the eighth column, entitled “Long-Term Outcome Measure,” are the following two bullet points: BRFSS, CHARS, and Vital Statistics data; and 2) CHARS and Vital Statistics datasets.

Planning for 2006–2007 improved previous years' efforts for several reasons. First, the planning process was a collaborative effort. Second, evaluation and surveillance data, collected as part of the previous years' evaluation efforts, were used in program planning efforts. Given the involvement of internal and external partners in the WaHDSPP, the program staff realized the potential benefits of the partners' input in planning program activities and evaluation efforts. As a result, partners and key stakeholders were invited to participate in the planning retreat for the 2006–2007 continuation application. The involvement of partners and stakeholders responsible for implementing program activities added depth and richness to the discussion. This collaborative process allowed for an in-depth discussion of the barriers to and facilitators of implementing program activities, factors that affect outcome attainment, and future program directions. These discussions were immensely helpful in developing work plans that linked to successful outcomes and that were both feasible and appropriate, given WaHDSPP resources.

The WaHDSPP program manager scheduled time during the planning retreat to review surveillance, evaluation, and assessment results from the previous year, ensuring that these data were taken into account in the proposed program activities. Involving partners and stakeholders in this process provided an opportunity to discuss results with people who were the most knowledgeable about the activities, which led to a better understanding of program activities by the entire planning committee and more realistic approaches to program improvement. In addition, being part of the program planning effort increased buy-in and "ownership" of the WaHDSPP. Once work plans were developed, the logic model and the LEP were updated to reflect proposed activities and outcomes. Collaborating on the logic model revisions provided new clarity in the short-, intermediate-, and long-term objectives for both staff and partners, and this clarity facilitated the selection of indicators.

The planning retreat for 2007–2008 focused on responding to the FOA released by the DHDSP and included newly hired WaHDSPP staff. As during the previous year, key partners and stakeholders were involved in planning, and evaluation and surveillance data were used to guide planning discussions. One new feature in the planning process involved categorizing proposed activities to align with the spectrum of prevention outlined in the Action Plan. The Action Plan, developed collaboratively by the DHDSP and its national partners, describes a comprehensive approach to addressing heart disease and stroke, from preventing risk factors to preventing recurrent cardiovascular events. The FOA encouraged states to use the Action Plan to guide development of their applications. By comparing proposed activities with the Action Plan, the WaHDSPP was able to identify a gap in its interventions, leading to the development of a new objective for the assessment of rehabilitation capacity.

**Figure 3.**
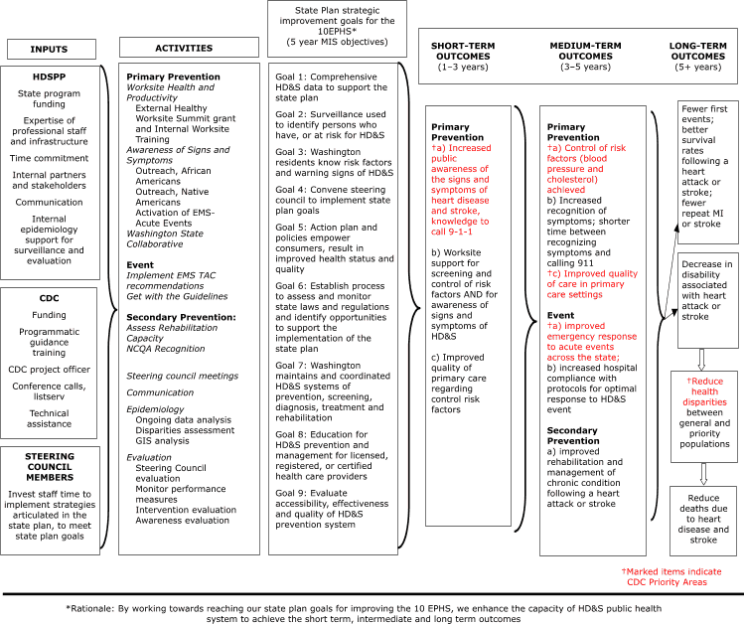
Washington State Heart Disease and Stroke Prevention Program (WaHDSPP) Logic Evaluation Plan, 2007–2008. (HDSPP indicates Heart Disease and Stroke Prevention Program; CDC, Centers for Disease Control and Prevention; EMS TAC, Emergency Medical Service Technical Advisory Committee; NCQA, National Committee for Quality Assurance; EPHS, essential public health services; MIS, management information system; HD&S, Heart Disease and Stroke; MI, myocardial infarction.)

Having additional program staff available to develop work plans for the application enabled partners to collaborate more closely than in years past to revise the logic model. The updated logic model (Figure 3) demonstrated the WaHDSPP's alignment with the Action Plan, categorizing activities and their outcomes as either primary prevention, acute event, or secondary prevention, and reflected more mature thinking on the part of the WaHDSPP about the specific changes expected as a result of program activities.

**Table:** 

The figure begins with a series of boxes organized into three columns. The three boxes arranged on top of each of the columns read 1) Inputs, 2) Activities, and 3) State Plan strategic improvement goals for the 10EPHS*. In the first column, entitled “Inputs,” are three boxes arranged from top to bottom. Inside the first box, entitled “HDSPP,” are the following bullet points: State Program Funding; Expertise of professional staff and infrastructure; Time Commitment; Internal Partners and Stakeholders; Communication; and Internal epidemiology support for surveillance and evaluation. Inside the second box, entitled “CDC,” are the following bullet points: Funding; Programmatic Guidance; Training; CDC Project Officer; Conference calls, listserv; and Technical Assistance. Inside the third box, entitled, “Steering Council Members,” is the following information: Invest staff time to implement strategies articulated in the State Plan, to meet State Plan Goals. All three boxes in the first column are connected with a right-pointing bracket to the boxes in the second column.
The second column, entitled “Activities,” contains one large box beneath the title box. The large box contains three major sections, each with information listed beneath. The three major sections are: 1) Primary Prevention, 2) Event, and 3) Secondary Prevention. Under the “Primary Prevention” section, three major areas are listed: 1) Worksite Health and Productivity, 2) Awareness of Signs and Symptoms, and 3) Washington State Collaborative. The “Worksite Health and Productivity” section lists the following two items: 1) External Healthy Worksite Summit, and 2) Grant and Internal Worksite Training. The “Awareness of Signs and Symptoms” section lists the following three items: 1) Outreach, African Americans; 2) Outreach, Native Americans; and 3) Activation of EMS-Acute Events. Under the “Event” major section, the following two items are listed: 1) Implement EMS TAC recommendations, and 2) Get with the Guidelines. Under the “Secondary Prevention” major section, six subheadings are listed: 1) Assess Rehabilitation Capacity, 2) NCQA Recognition, 3) Steering Council meetings, 4) Communication, 5) Epidemiology, and 6) Evaluation. Underneath the “Epidemiology” subheading, the following three items are listed: 1) Ongoing data analysis, 2) Disparities assessment, and 3) GIS analysis. Underneath the “Evaluation” subheading, the following four items are listed: 1) Steering Council evaluation, 2) Monitor performance measures, 3) Intervention evaluation, and 4) Awareness evaluation.
The third column, entitled “State Plan strategic improvement goals for the 10EPHS*,” contains one large box beneath the title box. The large box lists the following nine goals: 1) Comprehensive HD&S data to support the State Plan; 2) Surveillance used to identify persons who have, or at risk for HD&S; 3) WA residents know risk factors & warning signs of HD&S; 4) Convene Steering Council to implement State Plan Goals; 5) Action plan and policies empower consumers, result in improved health status and quality; 6) Establish process to assess and monitor State laws and regulations and identify opportunities to support the implementation of the state plan; 7) WA maintains and coordinated HD&S systems of prevention, screening, diagnosis, treatment and rehabilitation; 8) Education for HD&S prevention and management for licensed, registered, or certified HC providers; and 9) Evaluate Accessibility, Effectiveness and Quality of HD&S Prevention system.
To the right of column three are another series of three columns, each with a heading box above them. The first three columns already described are connected to the second series of three columns with a right-pointing bracket. The heading boxes above the second set of three columns read, “Supporting Outcomes (1-3 years),” “Medium-term Outcomes (3-5 years),” and “Long-term Outcomes (5 years +).” Underneath the column entitled “Supporting Outcomes (1-3 years)” is a box that contains the heading, “Primary Prevention,” followed by three bullet points: 1) Increased public awareness of the signs and symptoms of heart disease and stroke, knowledge to call 9-1-1 [noted as a CDC priority area]; 2) Worksite support for screening and control of risk factors AND for awareness of signs and symptoms of HD&S; and 3) Improved quality of primary care regarding control risk factors. The first column is connected to the second column with a right-pointing bracket.
Underneath the column entitled “Medium-term Outcomes (3-5 years),” is a box that contains three headings: 1) Primary Prevention, 2) Event, and 3) Secondary Prevention. Underneath the “Primary Prevention” heading are the following three bullet points: 1) Control of risk factors (blood pressure and cholesterol) achieved [noted as a CDC priority area]; 2) Increased recognition of symptoms; shorter time between recognizing symptoms and calling 911; and 3) Improved quality of care in primary care settings [noted as a CDC priority area]. Underneath the “Event” heading are the following two bullet points: 1) improved emergency response to acute events across the state [noted as a CDC priority area]; and 2) increased hospital compliance with protocols for optimal response to HD&S event. Underneath the “Secondary Prevention” heading is the following bullet point: improved rehabilitation and management of chronic condition following a heart attack or stroke. The second column is connected to the first two boxes of the third column with a right-pointing bracket and two arrows.
Underneath the column entitled “Long-term Outcomes (5 years +)” are four boxes arranged vertically. The first box contain the following information: Fewer first events; better survival rates following a heart attack or stroke; fewer repeat MI or stroke. The second box contains the following information: Decrease in disability associated with heart attack or stroke. The second box is connected to the third box with a downward-pointing arrow. The third box contains the following information: Reduce health disparities [noted as a CDC priority area] between general and priority populations. The third box is connected to the fourth box with a downward-pointing arrow. The fourth box contains the following information: Reduce deaths due to heart disease and stroke.
Underneath the six columns is the following text: “Rationale: by working towards reaching our state plan goals for improving the 10 EPHS, we enhance the capacity of HD&S public health system to achieve the short-term, intermediate, and long-term outcomes.”

A second feature of the 2007–2008 planning process involved the use of the CDC DHDSP's *Developing an Evaluation Plan* (4). This guide is one in a series developed by the DHDSP to assist states in their evaluation efforts. The WaHDSPP used the guide to systematically develop evaluation questions, leading to more well-rounded evaluation plans for individual activities and for the overall program (Table). The evaluation terms used by the program were revised to be consistent with those provided by CDC; the term *measures* was replaced with *indicators* and *medium-term outcomes *were referred to as *intermediate outcomes*. The evaluation plan replaced the LEP and provided more details on methods and data sources that would be used to answer key evaluation questions.

## Relationship Among Activities, Evaluation Plans, and the Logic Model

This section describes an outreach activity proposed for 2007–2008 and how it is being evaluated on the basis of the logic model. The African American Awareness and Screening Project takes place in barbershops and hair salons that have predominantly African American clients in two Washington counties with large African American populations. The project, which began in 2006, consists of training barbers and stylists to provide information about hypertension and the signs and symptoms of heart attack and stroke to their clients. Blood pressure readings are taken, and clients with high blood pressure are encouraged to see a health care provider.

### Links to the program logic model

As shown in the 2007–2008 logic model, the African American Awareness and Screening Project directly contributes to the supporting (i.e., short-term) outcome of increased public awareness of heart disease and stroke signs and symptoms. This activity is also linked to the intermediate outcomes of increased recognition of symptoms and reduced time between recognition of symptoms and taking action to call 911 and to greater control of risk factors (e.g., high blood pressure, elevated cholesterol levels), on the basis of the theory that increased awareness of high blood pressure and high cholesterol levels will lead to improved control of these risk factors.

### Evaluation approach

Evaluation planning focused on the stage of development of the activity. Because the African American Awareness and Screening Project was implemented in 2006, evaluation efforts targeted the implementation of the activity and assessment of the outcomes of increased public awareness of signs and symptoms of heart attack and stroke, as well as control of risk factors. Furthermore, the logic model assisted in developing the evaluation questions. By looking at the logic model to determine expected activities and outcomes, WaHDSPP staff were able to develop and prioritize the questions that the evaluation should answer, including the fidelity of the project, the impact of the project, and lessons learned and implemented.

The evaluation involves a mixed-methods approach, including both qualitative and quantitative data, and includes 1) the identification of facilitators of and barriers to the project through key stakeholder interviews, 2) a review of quarterly progress reports to evaluate fidelity to the original project plan, and 3) focus group sessions with participating barbers and stylists to evaluate trainings, project implementation, and perceptions about what worked and what did not work. Outcome evaluation will be conducted through review of screening results and follow-up to determine how many screened participants with high blood pressure were treated by a health care provider.

### Lessons Learned and Future Directions

Reflecting on the past 6 years of program activity, WaHDSPP staff identified several key lessons that have facilitated the use of programmatic logic models:

1. Logic models have assisted the WaHDSPP in developing its theory of change. By stating the theory of change, the program can better identify intermediate steps — and related indicators — that precede long-term outcomes and identify the incremental steps that precede short-term outcomes, allowing the program to monitor progress in a more proximal manner.
2. The WaHDSPP has used the annual occasion of preparing a continuing application as a time for collective reflection, not only on how to better refine existing activities or develop new ones, but also as a time to critically examine and improve the logic model and evaluation tools.
3. The logic model now categorizes activities and outcomes according to their place in the spectrum of prevention, allowing WaHDSPP staff to visually track the expected impacts resulting from program activities over time and to think more clearly about how achievements in impacts at one stage of prevention may facilitate achievements at another stage by shrinking the size of the vulnerable population (5).
4. Periodic reflection allows the program to incorporate new knowledge, such as information and resources from the CDC DHDSP, peers, and the literature to enhance evaluation planning.
5. Logic modeling is integral to program planning, implementation, and evaluation. The model set forth in CDC's six-step *Framework for Program Evaluation in Public Health* describes this collaborative approach (6).
6. Evaluation and logic model development should be conducted in partnership with program stakeholders, with results feeding directly back into ongoing program planning and progress monitoring. Logic models and evaluation plans are dynamic tools to guide the program in carrying out activities but should always be developed and refined in partnership with activity work plans and the key staff and partners involved in the work.

These lessons have been incredibly valuable in informing and improving the direction of the WaHDSPP and demonstrate how logic models are useful in public health program planning and evaluation.

## Figures and Tables

**Table.** Evaluation Plan^a^, Washington Heart Disease and Stroke Prevention Program (WaHDSPP)^b^, 2007–2008

Questions	Indicators	Data Sources	Data Collected by	Data Analysis	Communicating Results	

					To Whom?	How?
What key lessons were learned from evaluation of the previous barber/hair stylist outreach program conducted by WaHDSPP?	Product = List of key lessons learned	Written summary	July 2007–August 2007	Document review	CDC Steering Council Community partners in King and Pierce counties	Progress reports to funding agency (CDC) Calls with CDC Project Officer Present results to Steering Council Meeting with local partners
Was the hair stylist outreach program for community centers and faith-based organizations conducted as planned?	Process measures; feedback from stylists	Quarterly progress reports and stylist focus group	September 2007–February 2008	Analysis of focus group data		
What was the impact of the barber/hair stylist outreach program?	Of those whose screening results indicate high BP, the number who were treated by a health care provider	Interviews with sample of clients with high BP results	September 2007–February 2008	Analysis of survey responses		

CDC indicates Centers for Disease Control and Prevention; BP, blood pressure.

a Based on the CDC Division for Heart Disease and Stroke Prevention Evaluation Guide.

b Program promotes awareness of signs and symptoms of heart disease and stroke and targets African Americans (supporting outcomes: increased public awareness of risk factors, signs and symptoms of heart disease and stroke, and calling 911; intermediate outcomes: controlled risk factors, increased recognition of symptoms, and decreased time between recognizing symptoms and calling 911).
